# Infectiousness of Milk

**Published:** 1895-02

**Authors:** 


### REVIEW OF VETERINARY SANITARY WORK.

INFECTIOUSNESS OF MILK. Report of Dr. Harold C. Ernst to the Massachusetts Society for Promoting Agriculture. This valuable report, by Dr. Ernst, of Harvard University, with the collaboration of Drs. Peters, Jackson, and Frothingham, has just come to our notice, and we are certainly delighted with the evidence of the good work performed and the results obtained. This report represents many months of faithful and careful work. The investigations comprise: "1. A careful and persistent microscopic examination of the milk from cattle whose udders were apparently healthy. 2. Inoculation experiments with such milk. 3. Feeding experiments with same milk. 4. Similar investigations of the milk-supply of Boston. 5. The gathering of such evidence as possible from medical men and veterinarians as to cases of probable infection through tuberculous milk that had come under their observation." An experimental farm was provided where the animals were kept and the inoculation and feeding experiments carried on. The laboratory work was carried on at the Harvard Medical School. Statistics as to the evidence for and against the probability, from a clinical standpoint, of infection from milk, were obtained from many physicians and veterinarians in different parts of the country. The report is full of *facts* and interesting reading, and is certainly a relief from the mass of generalization in the literature of to-day upon the subject. Pity that other States would not carry on such work, and that the General Government would not appropriate a sum of money for the *investigation* of the subject of tuberculosis from every standpoint.

